# Recent advances in bacteria-mediated cancer therapy

**DOI:** 10.3389/fbioe.2022.1026248

**Published:** 2022-10-12

**Authors:** Shuya Liang, Chao Wang, Yingchun Shao, Yanhong Wang, Dongming Xing, Zhongmin Geng

**Affiliations:** ^1^ Department of Dermatology, The Affiliated Hospital of Qingdao University, Qingdao, China; ^2^ Qingdao Cancer Institute, The Affiliated Hospital of Qingdao University, Qingdao, China; ^3^ School of Life Sciences, Tsinghua University, Beijing, China

**Keywords:** cancer therapy, bacteria, tumor microenvironment, tumor targeting, combination therapy

## Abstract

Cancer is among the leading cause of deaths worldwide. Although conventional therapies have been applied in the fight against the cancer, the poor oxygen, low extracellular pH, and high interstitial fluid pressure of the tumor microenvironment mean that these treatments fail to completely eradicate cancer cells. Recently, bacteria have increasingly been considered to be a promising platform for cancer therapy thanks to their many unique properties, such as specific tumor-targeting ability, high motility, immunogenicity, and their use as gene or drug carriers. Several types of bacteria have already been used for solid and metastatic tumor therapies, with promising results. With the development of synthetic biology, engineered bacteria have been endowed with the controllable expression of therapeutic proteins. Meanwhile, nanomaterials have been widely used to modify bacteria for targeted drug delivery, photothermal therapy, magnetothermal therapy, and photodynamic therapy, while promoting the antitumor efficiency of synergistic cancer therapies. This review will provide a brief introduction to the foundation of bacterial biotherapy. We begin by summarizing the recent advances in the use of many different types of bacteria in multiple targeted tumor therapies. We will then discuss the future prospects of bacteria-mediated cancer therapies.

## Introduction

Cancer is one of the most devastating diseases worldwide and is estimated to pose a serious threat to more than 26 million people by 2030 (Geng et al., 2021b). Although conventional therapies (e.g., surgery, chemotherapy, radiation therapy, and immunotherapy) are still the standard treatment in the management of cancer for many patients, these treatments have failed to completely eradicate cancer cells (Patyar et al., 2010; Sedighi et al., 2019; Geng et al., 2021c). For instance, surgery can easily take a solid tumor out of the body, but it cannot be effective for subclinical metastases and cancer recurrence (Wyld et al., 2015). Chemotherapy, a noninvasive cancer treatment method, can effectively inhibit the growth and metastasis of tumor cells by spreading chemical drugs throughout the whole body. However, the poor selectivity of chemotherapy drugs for tumor cells over normal tissue results in serious toxic and side effects (Pérez-Herrero and Fernández-Medarde, 2015). Therefore, alternative or complementary therapies—including photodynamic therapy (PDT), gene therapy, and biotherapy—have been proposed to enhance the effectiveness of cancer therapy (Geng et al., 2021a; Belete, 2021; Zhang et al., 2022). As early as the nineteenth century, Willhem Busch and William Coley independently discovered that live bacteria could successfully cause regression in tumors (Busch, 1868; Coley, 1991). Although the precise mechanism of the regression process is not well determined, it is considered that the tumor microenvironment (TME) may provide a more favorable condition for bacterial growth. These early successes encouraged multiple investigations of bacterial-based tumor therapies. However, despite their antitumor activity, the treated outcomes remained unstable. In contrast some bacterial species can contribute to cancer’s progression by creating a proinflammatory microenvironment, influencing the autophagy, or suppressing immune cell function (Li S. et al., 2021). Consequently, antibacterial therapies that inhibit the growth of tumorigenic bacteria will be beneficial to cancer therapy. More recently, bacteria, as drug-delivery vehicles and immune modulators in tumor biotherapy, have attracted increasing attention with the progress of biotechnology and immunology thanks to their unique properties, including disease-site localization and therapeutic agent production (Chen et al., 2021; Pan et al., 2021; Kang et al., 2022).

**FIGURE 1:**
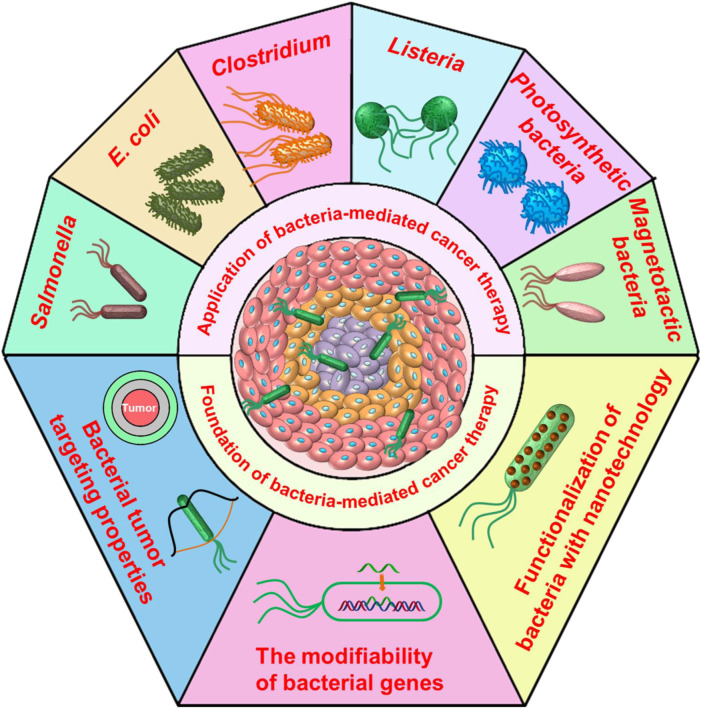
Schematic representation of the foundation and application of bacteria used for cancer treatments.

As our understanding of TME improves, uncontrolled cell proliferation, metabolic abnormalities, and aberrant neovascularization result in some unique features in TME, such as low-oxygen tension or hypoxia, high interstitial fluid pressure, and low extracellular pH, and can finally fuel cancer’s progress, treatment resistance, and immunosuppression (Fukumura et al., 2018; Lin et al., 2021). However, the hypoxic TME are conducive to some specific bacteria migrating to the region of the solid tumor, which can be attributed to their self-propulsion ability and anaerobic properties (Yin et al., 2022). In addition, as a result of their unique capabilities, bacteria show great potential as promising diagnostic and therapeutic agents. First, a remarkable feature of bacteria is that their genetics can be simply modified to produce and release specific compounds, which can combine with the production of specific toxins to stimulate an effective immune response, which leads to tumor regression (Forbes, 2010; Danino et al., 2015; Kucerova and Cervinkova, 2016; Xu et al., 2018). Second, different bacterial species can colonize various body sites of mammalian hosts, such as the skin, the urogenital system, and the gut, as well as disease sites (e.g., abscesses and tumor tissues) (Talib and Mahasneh, 2012; Fan et al., 2018; Su et al., 2022). In particular, the inherent tropism of bacteria for different types of tumors and their ability to overcome the vascular barrier means that they have potential for use in tumor-targeting delivery (Wu et al., 2021). Furthermore, the cell surfaces of bacteria can be decorated with organic or inorganic materials, including various types of nanomaterial and therapeutic agents for cargo delivery (Zhu C. et al., 2021; Ye et al., 2021; Jiménez-Jiménez et al., 2022). Additionally, bacterial derivatives—including outer membrane vesicles (OMVs), minicells, and bacterial protoplast-derived nanovesicles—have been shown to have great potential as safe and efficient drug carriers (Ali et al., 2020). These comparative advantages have aroused interest in the field of biotherapy over the past few years. To date, several types and strains of bacteria (e.g., *Clostridium* spp., *Salmonella* spp., *Escherichia coli* (*E. coli*) spp. and *Listeria* spp.) have been found to be well suited as anticancer agents (Rong et al., 2020). In this review, we will briefly update the most recent developments in bacteria for biotherapy (Figure 1). We then investigate some of the different strategies that have been developed to enhance the safety and efficiency of bacterial functionalization for biotherapy. Finally, we discuss the challenges and prospects for bacteria-based cancer treatments.

## Foundation of bacteria-based cancer treatments

### Bacterial tumor-targeting properties

**FIGURE 2:**
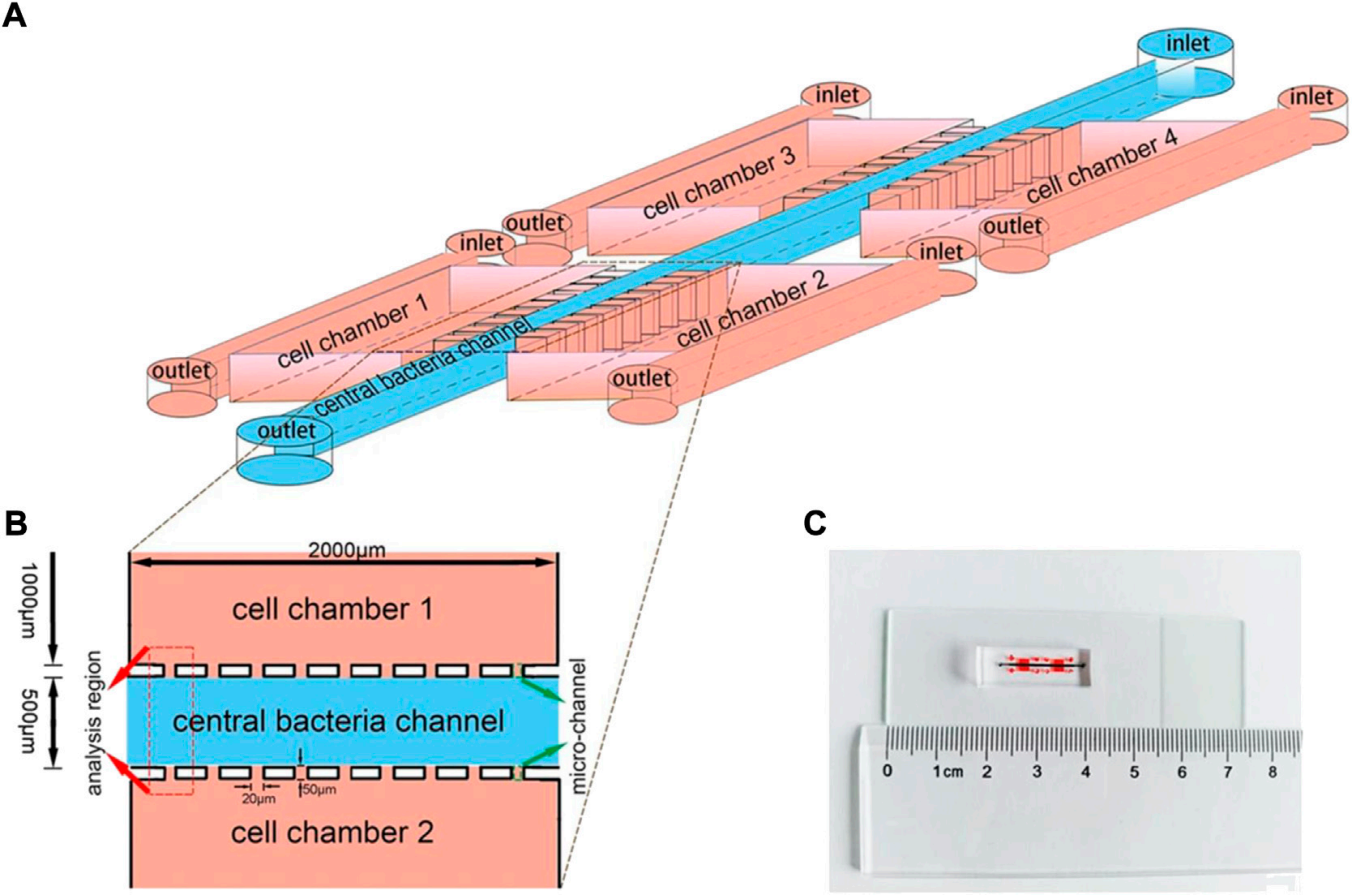
Schematic depiction of a microfluidic device. **(A)** An illustration of the device for investigating bacterial chemotaxis mechanism. Microfluidic device constructed by central bacteria channel, four separate cell-culture chambers, and micro-channels. **(B)** The analysis region used to quantify the preferential accumulation of bacteria sited between two chambers. **(C)** An image of the integrated microfluidic device. Reproduced with permission from Song et al. (2018). Copyright 2018, Springer Nature.

It is generally accepted that the specific capacity of bacteria to target solid tumors can be attributed to both passive and active mechanisms (Lou et al., 2021). It is also assumed that live bacteria may first enter a solid tumor by accidental entrapment in the leaky vasculature. They then gather in the tumor due to the hemorrhage that is caused by bacterial infection (Lee, 2012). The active mechanisms of bacteria in TME differ depending on oxygen tolerance, which likely include certain signal molecules and chemotactic factors generated by dying tumor cells and poor-oxygen microenvironment (Lee, 2012). Obligate anaerobes (e.g., *Bifidobacterium* and *Clostridium*) cannot survive in an oxygen-enriched environment, thus further favoring migration toward the anoxic regions of tumors. In fact, a fully deoxygenated microenvironment is unique to tumors and hardly occurs in most normal tissues of the body, which results in obligate anaerobes effectively accumulating in the regions of tumors (Dang et al., 2001). Malmgren and Flanigan (1955) demonstrated this specificity and showed that only tumor-bearing mice die from infection when injecting *Clostridium*. Facultative anaerobes—including *Pseudomonas*, *Escherichia* and *Salmonella*—target and accumulate in a solid tumor with more complex mechanisms, including: 1) bacteria are entrapped in the chaotic and tortuous vasculature of a tumor, 2) the limited blood circulation protects bacteria from clearance by the immune system, 3) bacteria accumulate in tumor tissue following inflammation, 4) bacteria preferentially proliferate in TME, and 5) bacteria are attracted to compounds produced by tumor (Forbes et al., 2003; Kasinskas and Forbes, 2006, 2007; Leschner et al., 2009). Song et al. (2018) designed a microfluidic device to investigate the role of several signal molecules in tumor targeting (Figure 2). They find that *E. coli* prefer to colonize in tumor tissue, which secretes compounds such as serglycin, TGF-β2, and clusterin. Additionally, the nutrient-rich environment of tumors is uniquely attractive to bacteria. Stritzker et al. (2007) showed that some facultative anaerobes (e.g., *Escherichia* and *Salmonella*) are able to sense the nutrient-rich region in and around the tumor center, which results in enhanced accumulation in tumors. Actually, these mechanisms are neither mutually exclusive nor completely independent, and bacteria might activate multiple pathways to specifically target tumors (Garza-Morales et al., 2020). Certainly, different strains may exhibit distinct mechanisms lead to different tumor-targeting abilities. Furthermore, motility, as a basic feature of bacteria, facilitates bacteria to rapidly penetrate into the tumor tissues like “biorobots.”

### The modifiability of bacterial genes

The main limitations of bacteria-based cancer treatments are the potential pathogenicity and toxicity of bacteria to human cells and organs. Another important characteristic of bacteria is that they are easy to control using genetic modifiability, which means that they can be modified into versatile platforms using methods of synthetic biology. Generally, various plasmids for expressing therapeutic proteins, anticancer agents, or tumor-specific antigens are transformed into bacterial cells to potentiate the antitumor activity and reduce the systemic toxicity (Jiang et al., 2010; Zhang et al., 2014; Zhou et al., 2018). To reduce the inherent pathogenicity of *E. coli*, *Salmonella*, *Listeria*, and *Clostridium*, it is usually necessary to delete virulence factor genes for cancer therapy (Felgner et al., 2016a; Felgner et al., 2016b). Low et al. (1999) reported that removing the myristoylation of lipid A results in low expression of tumor necrosis factor α by disrupting the msbB gene of *Salmonell*a and *E. coli*. The lipopolysaccharide content of *Salmonell*a obviously decreases when deleting the msbB gene and the safety of this endotoxin-reduced *Salmonella* for patients has been demonstrated in phase I clinical trials (Toso et al., 2002). Quintero et al. (2016) engineered the *Salmonella* strain VNP20009 to highly express epidermal growth factor receptor-targeted Pseudomonas exotoxin A. This approach shows significant antitumor activity against epidermal growth factor receptor-positive tumor cells. However, bacteria can locate in other normal organs for a short time when beginning to enter the body. However, the consistently expressing cytotoxic drugs may cause toxicity in healthy tissue. Therefore, the precise control of bacterial therapeutic gene expression at specific times and locations can enhance the concentration of cytotoxic drugs in a solid tumor and reduce the systemic toxicity (Din et al., 2016). Tumors may be treated more accurately by designing an expression system, which will respond sensitively to endogenous or exogenous stimuli. For example, hypoxia TME has been used to design inducible promoters to anticancer peptides, gene fragments, and proteins expression in bacteria for specific delivery to tumors (Chen Y. et al., 2020). Zhao et al. (2005) designed an auxotrophic *Salmonella* strain, which prefer to colonize in tumor tissue 2000-fold higher than in healthy tissue, owing to abundant leucine and arginine in the tumor. By modifying bacterial genes, synthetic biology has been able to endow bacteria with the ability to be triggered by the expression of different effector proteins under different stimulation for antitumor therapies.

### Functionalization of bacteria with nanotechnology

**FIGURE 3:**
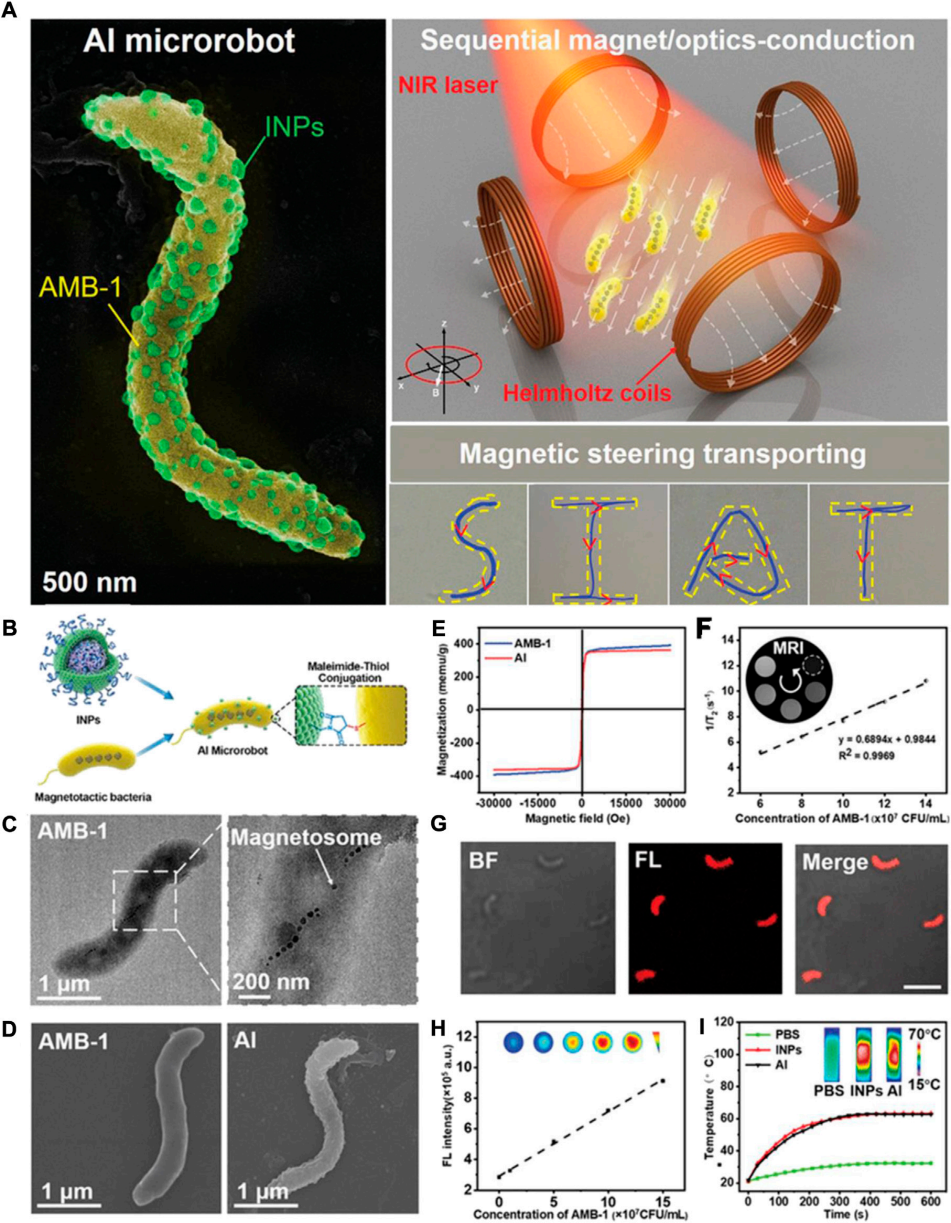
Design, preparation, and characterization of a biohybrid microrobots. **(A)** Schematic illustration of biohybrid microrobots (left: representative SEM image of a biohybrid microrobot; right: illustration of the biohybrid microrobot under sequential magnet/optics-conduction; bottom: the transporting trajectories of biohybrid microrobot under complicated 2D magnetic steering.) **(B)** Magnetospirillum AMB-1 was facilely coated by nanophotosensitizer INPs to construct an on-demand controllable AI microrobot. **(C)** Representative SEM image of AMB-1 (right: magnetosome chains inside AMB-1). **(D)** Representative SEM image of AMB-1 and AI microrobot. **(E)** Magnetic hysteresis curves of the AI microrobots and AMB-1 at 300 K. **(F)** Relaxation ratios of the AI microrobots solution. **(G)** Representative fluorescence confocal image of AI microrobots. Scale bars: 5 µm. **(H)** NIR fluorescence intensity and corresponding images of the AI microrobots solution. **(I)** Light-triggered curve of PBS, nanophotosensitizer INPs and AI microrobots under 808 nm laser 10 min. Reproduced with permission from Xing et al. (2021). Copyright 2020, John Wiley & Sons.

Compared to mere bacterial genetic modifications, another obvious advantage of bacteria is the easy functionalization of the intracellular and cell surface, which will lead to multiple synergistic cancer treatments. Functionalized bacteria take full advantage of bacterial properties—including motility, low-oxygen growth conditions, and environmental sensitivity—to achieve accurate delivery and controllable release of drugs for improving antitumor efficacy. For example, bacteria can be designed as micro- or nanorobots, which show excellent biocompatibility and high drug loading efficiency for potential clinical translation. In addition, functionalized bacteria have several unique advantages when used as drug-delivery systems, including longer persistence in the blood circulation, preferential accumulation in the tumor, selective drug loading, and controlled release (Yin et al., 2022). Some functionalized bacteria can even be activated by in vitro signals (e.g., thermal, ultrasonic, or magnetic signals). Xing et al. (2021) prepared a biohybrid drug-delivery system that uses unique magneto-driven and light-triggered AI microrobots for active tumor-targeted therapy (Figure 3). In this system, AI microrobots (i.e., *Magnetospirillum magneticum*) provide the ability to target the tumor tissue via an external applied magnetic field, and indocyanine green (ICG) nanoparticles are used as imaging and photothermal therapy (PTT) agents. Additionally, bacteria-mediated biosynthesis of nanoparticles for cancer therapy has been considered to bring a new vitality in the area of cancer treatments. The combination of nanotechnology and bacteria is a promising drug-delivery system for cancer therapy.

## Applications of bacteria-mediated strategies in cancer therapy

Recently, a large number of studies on bacteria-mediated cancer therapy have been reported. Numerous bacteria species have been used, not only to express specific therapeutic proteins or anticancer agents but also to achieve targeted drug delivery, which then directly kills the cancer cells. Moreover, the combination of bacteria-mediated therapy and conventional therapy can lead to multiple synergistic antitumor strategies, which can cause more efficient tumor regression and can help in the development of novel antitumor strategies with systemic safety and effectiveness. The bacteria species that have been used in mediated antitumor treatments are described and discussed in the following subsections.

### *Salmonella*-mediated cancer therapy

*Salmonella*, a gram-negative bacterium, are facultative anaerobic bacteria, which have been demonstrated to be a cancer-targeting therapeutic (Gupta et al., 2021). However, the applications of *Salmonella* have the potential to cause severe side effects, mainly attributed to the control of the endotoxin lipid A by *msb*B gene (D’Hauteville et al., 2002). The virulence of *Salmonellae* can be decreased using a gene knock-out strategy to get attenuated *Salmonellae*, which still preserve their high affinity to solid tumors. It has been reported that attenuated *Salmonellae* can penetrate into deep regions of tumors, where are generally considered to be unreachable by conventional therapeutic drugs (Li T. et al., 2021). Therefore, attenuated *Salmonellae* are regarded to be a promising candidate for bacteria-mediated cancer therapy thanks to their ability to specifically target tumors, rapid migration speed, and intrinsic toxicity (Luo et al., 2016). To date, a variety of *Salmonella* strains combined with other therapy strategies have been shown to successfully inhibit tumor growth.

**FIGURE 4:**
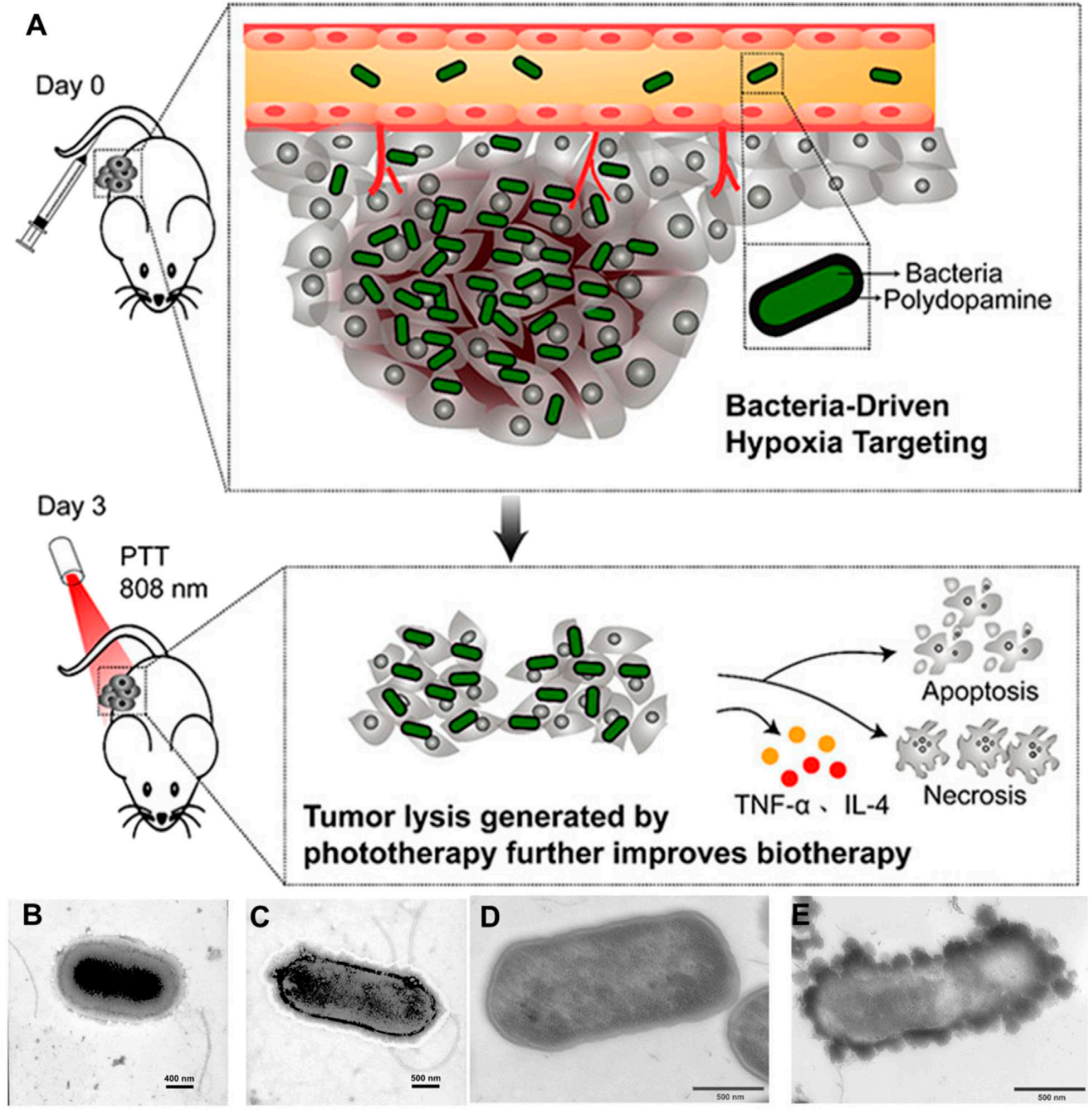
Illustration, preparation, and characterization of pDA-VNP for cancer therapy. **(A)** Schematic depiction of pDA-VNP for combined biotherapy and photothermal therapy. Representative TEM image of VNP20009 **(B)** and pDA-VNP **(C)**. Representative TEM image of the microtome-sliced VNP20009 **(D)** and pDA-VNP **(E)**. Reproduced with permission from Chen et al. (2018). Copyright 2018, American Chemical Society.

PTT has emerged as a great potential cancer treatment, which commonly depends on photothermal agents (PTA) to absorb and convert near-infrared (NIR) light into heat to ablate tumor cells with minimal invasiveness and high specificity. However, the poor penetration of PTA and the immunosuppressive TME protects residual tumor cells from fighting against T cells, thereby inducing recurrence and metastasis. Yi et al. (2020) discovered that attenuated *Salmonella* will proliferate in tumor tissues after intravenous injection, while they rapidly clear in normal organs. Interestingly, bacteria-induced inflammation triggered the formation of thrombosis by destroying tumor blood vessels in the solid tumors. Various types of solid tumors had been observed to turn into an obvious blackish color with strong NIR absorbance, displaying potential for PTT. Under laser irradiation, the bacterial-infected solid tumors were efficiently ablated. Subsequently, PTT triggered antitumor immune responses validly suppressed the tumor metastasis and recurrence. The safety and antitumor activity of attenuated *Salmonella* VNP20009 has been examined in a phase 1 clinical trial. Unfortunately, the insignificant antitumor effects and dose-dependent toxicity caused the trial to terminate (Toso et al., 2002). Chen et al. (2018) reported a combination of the tumor-targeting ability of *Salmonella* VNP20009 and polydopamine (PDA) mediated PTT to achieve superior anticancer effect (Figure 4). Engineered bacteria were decorated with PDA on the bacterial surface via oxidation and self-polymerization, which were injected via a tail vein into tumor-bearing mice. As bacteria and as photothermal therapeutic agents, PDA-coated *Salmonella* VNP20009 could target the hypoxic regions of tumor tissue, which will eliminate the tumor cells without relapse or metastasis with a single injection and NIR irradiation. Because most of PTA is difficult to penetrate into the center of solid tumors, large tumor (≈200 mm^3^) treatments are still challenging. Chen W. et al. (2020) successfully encapsulated on the surface of *Salmonella* VNP20009 with PDA to treat large size of melanoma tumors by combining bacterial therapy, PTT, and immunotherapy. In this study, bacteria, which carry PTA, are intravenously injected and can efficiently achieve deep tumor hypoxic regions, followed by NIR irradiation at the tumor site. The photothermal-induced dead tumor cells produce tumor cell lysates that can activate the body’s immune response. Meanwhile, the tumor cell lysates can be used as a nutrient for the reproduction of bacteria in solid tumors. To apply this approach to treat a large tumor, the authors combine anti-programmed cell death-1 peptide to change the tumor immunosuppressive microenvironment to enhance the antitumor effects. This triple-combined therapy provides a fresh strategy for antitumor treatments. Moreover, Mi et al. (2021) covalently attached ICG nanoparticles to the surface of *Salmonella Typhimurium* YB1 (YB1-INPs) with amide bonds for large tumor precision therapy. This treatment strategy benefited from tumor cell destruction and the generation of bacteria-attracting nutrients after PTT, with 14-fold bioaccumulation of YB1-INPs than no photothermal intervention.

Chemotherapy is the first-line treatment for cancer patients and has been extensively applied to against various solid tumors over the past few decades (Cao and Liu, 2020). However, the standard anticancer chemotherapeutic agents—including doxorubicin, paclitaxel, camptothecin, carboplatin, and cisplatin—do not have the ability to target cancer cells, which directly results in insufficient dosage to tumor areas and decreases the treatment efficacy. In addition, non-specific distribution of these chemotherapeutic agents can cause undesirable severe cytotoxic effects by normal tissues enrichment and damage. Therefore, the development of a safe and efficient system to deliver chemotherapeutic agents for tumor-targeting therapy is urgent required. Nguyen et al. (2016) proposed a new bacteria-mediated microrobot (bacteriobot), which combines tumor-targeting *Salmonella Typhimurium* with paclitaxel-loaded liposome. This bacteriobot was prepared by binding drug-loaded liposomes to biotin molecules displayed on the surface of the bacteria, which displays a better therapeutic effect than conventional drug-loaded liposomes. Ektate et al. (2018) constructed thermobots by binding low-temperature sensitive liposome on the outer membrane of attenuated *Salmonella*, which was applied to colon cancer chemo-immunotherapy. The thermobots released doxorubicin from liposomes by ultrasound heating at 40–42°C after reaching the tumor sites.

In recent years, *Salmonella* have been modified not only with photo-, chemo- and immuno-agents on their surface but also with polymer and metal nanomaterials, serving as a reliable “mailman” to transport drug-loaded cargo though membrane attachment to tumor sites. Suh et al. (2019) developed a nanoscale bacteria-enabled autonomous drug-delivery system (NanoBEADS), which is prepared by binding poly (lactic-co-glycolic acid) nanoparticles to the surface of tumor-targeting *Salmonella Typhimurium* VNP20009. The NanoBEADS platform successfully evaluated the invasion of cancer cells, in vitro intratumoral transport, and in vivo distribution of mammary tumor mode. The predominant mechanisms of bacterial intratumoral penetration were attributed to the intercellular proliferation and translocation, which offered promising approaches for increased targeted delivery and interstitial biodistribution of nanotherapeutics. Although cancer immunotherapy has been considered to be the fourth major therapy, the inability of immuno-drugs to turn the “cold” tumor to “hot” has limited their use in treatments. Considering the immunosuppressive TME, the insufficient expression of tumor-associated antigens after conventional therapies meant that it was difficult to elicit immune responses (Chen et al., 2022). Wang et al. (2022) have recently shown that attenuated *Salmonella* VNP20009 camouflaged with cationic polymer nanoparticles can accumulate negatively charged tumor-associated antigens at the tumor’s periphery following radiotherapy. This approach improved the crosstalk between tumor-associated antigens and dendritic cells (DCs), and increased the activated ovalbumin-specific DCs, with the extended survival of tumor-bearing mice. Furthermore, the surface modification strategy can be used to construct DNA vaccines, producing efficient and versatile vaccines for cancer immunotherapy. Hu et al. (2015) reported a new bacterial vector, which was prepared by coating synthetic β-cyclodextrin-PEI600 nanoparticles on live attenuated *Salmonellae* to deliver oral DNA vaccines. Their results suggest that coating live bacteria with nanomaterials is a potential approach to design efficient and versatile DNA vaccines for cancer treatments. Taken together, *Salmonella*-mediated cancer therapy has encouraged researchers to further investigate the combination of *Salmonella* and other therapies to generate more effective cancer treatments.

### *E. coli*-mediated cancer therapy

*E. coli* are a gram-negative facultative anaerobic bacterium that belongs to the natural intestinal probiotic and has inherent anti-inflammatory properties. Like *Salmonella*, *E. coli* has the ability to target the hypoxic areas of a tumor after intravenous injection, while bacteria are rapidly eliminated in normal tissue due to the sensitivity of lipopolysaccharide on bacterial membrane to serum (Jiang et al., 2010). Additionally, the manipulation of gene expression can be convenient achieved by synthetic biology because the full genome of *E. coli* has been sequenced (Garamella et al., 2016). To meet the many different requirements of cancer therapy, several types of therapeutic strains have been engineered, including *E. coli* MG1655 (Kang et al., 2020), DH5α(Dai et al., 2021) and Nissle 1917 (EcN) (Yu et al., 2020). To date, *E. coli* has been used for a variety of applications in cancer treatment, displaying great promise.

**FIGURE 5:**
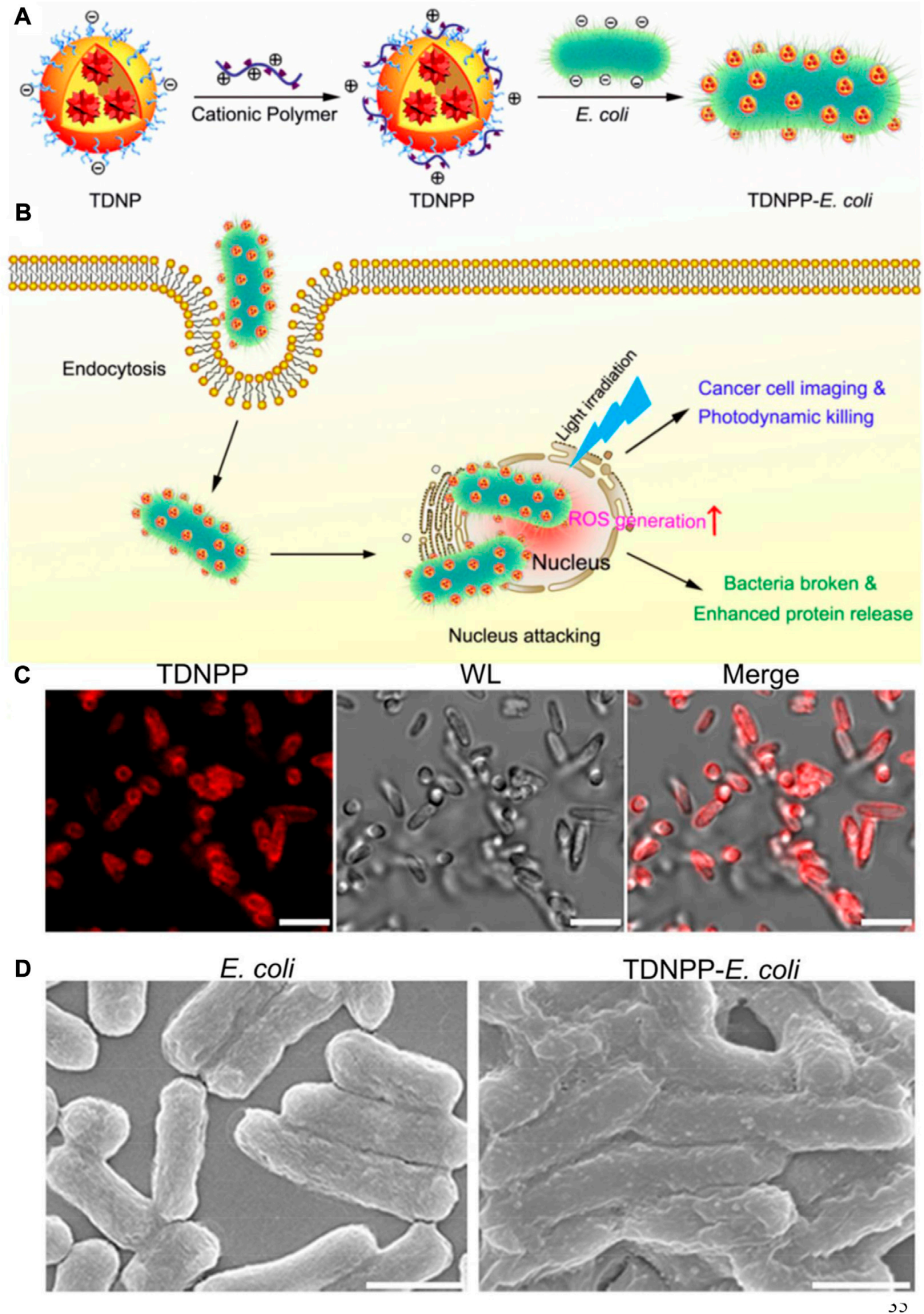
Schematic depiction of live bacteria carrying TDNPP for bacteria-mediated cancer therapy. **(A)** Illustration of the preparation procedure of TDNPP-*E. coli*. **(B)** Intracellular transport of TDNPP-coated live bacteria. **(C)** Representative confocal microscopy images of TDNNP-*E. coli*. Scale bar: 2 μm. **(D)** Representative SEM images of *E. coli* and TDNPP-coated *E. coli*. Scale bar: 1 μm. Reproduced with permission from Wu et al. (2019). Copyright 2019, American Chemical Society.

During the past few decades, PDT, as a noninvasive strategy for treating esophageal tumors, skin tumors, and lung tumors in the clinic with light, photosensitizer (PS) and oxygen, has been regarded as an extremely promising for high-efficient cancer therapeutic modality (Yang et al., 2022). However, the efficacies of PDT are far from satisfactory because PS poorly accumulates and can hardly penetrate tumor sites. Encouraged by the achievements of synthetic biology, bacteria with inherent tumor colonizing properties have been rationally designed for enhanced bacteria-mediated cancer combination therapy (Chowdhury et al., 2019; Chen Q. W. et al., 2020). Black phosphorus (BP), with high photothermal conversion efficiency and singlet oxygen generation efficiency, may potentially be applied in biomedicine. Ding et al. (2021) constructed a novel engineered bacterial system, which prepared by binding BP quantum dots (BPQDs) to the surface of *E. coli*, generating an *E. coli*/BPQDs system. The *E. coli* engineered to express catalase served as a generator of reactive oxygen species (ROS) from hydrogen peroxide under NIR irradiation. The preclinical simultaneous utility of biotherapy, PDT, and gene therapy was first proved to strengthen the antitumor effects. Dai et al. (2021) prepared a new bacteria-based drug-delivery system (*E. coli*@ZnPc-IR710) to improve the efficacy of zinc phthalocyanine (ZnPc) PS in PDT. The adhesion properties of a series of ZnPc PS with different charges on the cell wall of bacteria were investigated to define the highest loading capacity. With the help of bacteria, in vitro and in vivo studies have shown that *E. coli*@ZnPc-IR710 displayed a significantly enhanced inhibition of tumor growth compared with ZnPc-IR710. Similar research was conducted by Deng et al. (2021), in which *E. coli* was genetically modified to overexpress catalase for catalyzing H_2_O_2_ decomposition in solid tumors. PS chlorin e6 (Ce6) was coated by PDA for attachment on the surface of bacteria, which are used for tumor-targeted delivery, and consequently combined treatment of PTT and PDT. Directly introducing specific therapeutic genes and proteins into tumor cells for the cancer therapy by bacteria has drawn widespread interest. However, the release of therapeutic agents from bacteria inside cancer cells still remains difficult. Recently, Wu et al. (2019) reported a novel biohybrid system that was constructed by surface modification of *E. coli* with aggregation-induced emission (AIE) PS nanoparticles (TDNPP) for imaging and ablating tumor cells (Figure 5). The TDNPP coating on the membrane of bacteria not only facilitated *E. coli* into invade cancer cells but also generated ROS under NIR irradiation, leading to efficient release of therapeutic protein for cancer treatments. AIE-based PDT is a promising approach for cancer therapy but is still limited by many factors, such as poor laser penetration and hypoxia TME. To address these limitations, Zhu D. et al. (2021) developed a new bacteria-mediated hybrid AIEgen system (AE) to facilitate the hypoxia-tolerant PDT of colon tumors in situ via an interventional method. They synthesized an AIE PS conjugative molecule with two positive charges to generate singlet oxygen, which was delivered by bacteria and accumulation in orthotopic colon tumors for localized aggregation and PDT. Their in vivo results suggest that this was the first interventional PDT approach to successfully treat tumors, providing the potential to develop a hybrid AIEgen system. Alternatively, ROS can be generated by enzyme catalyzation in a solid tumor. Synthetic biology has been applied in the genetic engineering of bacteria for the preparation of ROS-generating enzymes. Fan et al. (2019) reported an engineered bacterium *E. coli* MG1655 that was genetically modified to overexpress the respiratory chain enzyme II, which could transfer electrons to free oxygen to generate H_2_O_2_. Subsequently, Fe_3_O_4_ nanoparticles were covalently conjugated to the surface of *E. coli* for initiation of Fenton-like reaction. In this design, engineered *E. coli* sustainably generated H_2_O_2_ to cause a Fenton-like reaction with producing toxic hydroxyl radicals for tumor apoptosis.

Another typical application of *E. coli* as an antitumor medicine is the delivery of chemotherapeutic drugs for cancer therapy. Xie et al. (2017) reported a dfferent strategy of directly linking doxorubicin (DOX) onto the cell wall of motile *E. coli* bacteria via acid-labile linkers (EcN-ca-Dox). In vitro experiments displayed that the viability of the bacteria was not significantly affected by drug conjugation, maintaining the bacterial motion velocity over 9 μm/s. The in vivo results demonstrated that the accumulation of DOX in the tumor was increased by intravenous injection of EcN-ca-Dox, thereby improving the temporal and spatial control of chemotherapeutic agent action. Off-target toxicity has been one of the most common drawbacks of chemotherapy. To address this problem, Hosseini-Giv et al. (2021) recently reported a new method named as bacterial directed enzyme-prodrug therapy (BDEPT), which used *E. coli* as enzyme carriers for prodrug conversion in the tumor site. They presented an engineering *E. coli*, which carried luxCDABE gene cluster and overexpressed β-glucuronidase (prodrug activating enzyme) for enzyme expression and luminescent emission. Glycyrrhizic acid, as a prodrug, could be converted to glycyrrhetinic acid (a potent anticancer agent) via enzyme expression. The results of their experiments demonstrate that the growth of induced 4T1 tumors in BALB/c mice was significantly delayed by BDEPT treatment. High-intensity focused ultrasound (HIFU) is a noninvasive therapy that has the potential to ablate tumors and facilitate controlled drug release in tumor tissue, and has already been applied for cancer therapy (Fite et al., 2021). However, the low thermal energy deposition and poor tumor targeting at the target site have restricted the widespread application of HIFU. Du et al. (2022) successfully constructed drug-loaded nanoparticles conjugated with genetically modified bacteria to facilitate HIFU ablation. Cationic lipid nanoparticles loading paclitaxel (PTX) and perfluorohexane could attach to the negatively charged surface of genetically engineered *E. coli* with gas vesicles via electrostatic adsorption, and subsequently targeted delivered to the tumor site. Under HIFU irradiation, the gas vesicles and perfluorohexane could be used as cavitation nuclei to improve the HIFU effect and released the PTX in the solid tumor, which displays the potential therapeutic efficacy of HIFU/chemo-synergistic treatment. However, the proinflammatory ability of bacteria-induced antitumor immune response is not enough to inhibit the growth of a tumor but when combined with chemotherapy may achieve high efficiency of tumor ablation. Sun et al. (2021) proposed a new strategy to prevent tumor metastasis and relapse by tandem-amplifying ROS-immunity responses. The chemo-drug doxorubicin (DOX) was conjugated onto the cell wall of glucose dehydrogenase (GDH)-overexpressed *E. coli* via bio-condensation reaction to enhance the antitumor efficacy. Nicotinamide adenine dinucleotide phosphate (NADPH) is generated by GDH-overexpressed *E. coli* and could promote the production of toxic ROS. Furthermore, DOX activated the membrane-spanning NADPH oxidases, which results in a substantial improvement of the ROS concentrations by catalyzing the conversion of NADPH and oxygen. Importantly, the both-in-one hybrid bacteria significantly potentiated the systemic antitumor immune responses and obviously suppressed tumor metastasis in both primary and distant tumor growth. In contrast from the both-in-one hybrid bacteria strategy, Wei et al. (2021) developed DOX-induced immunogenic cell death (ICD) to improve the efficacy of immunotherapy based on tumor-associated macrophage (TAM) polarization. In this study, the chemotherapeutic drug DOX-loaded poly (lactic-co-glycolic acid) were adhered to the surface of *E. coli* MG1655 though electrostatic absorption to fabricate a nanoparticle/bacteria complex (Ec-PR848) for targeted delivery and TAM polarization. Ec-PR848 could obviously accumulate in the tumor tissue and could effectively transform M2 macrophages to M1 macrophages. When combined with DOX-induced ICD, it could also enhance the efficacy of immunotherapy.

Similar to the description of *Salmonella*, *E. coli* plays an essential role in combination cancer treatment of bacteria-mediated therapy and PTT. Wang et al. (2019) showed that *E. coli* could selectively reduce perylene diimide derivative based supramolecular complex (CPPDI) in hypoxic tumor for precise PTT. Driven by the hypoxia TME, the bacteria first preferred to accumulate and replicate in the deep region of the tumor. They then reduced the CPPDI delivered by metalloproteinase-responsive liposome to convert to radical anions to facilitate PTT in the tumor, which decreased the potential side effects to normal tissue. The authors used both hypoxia tropism and intrinsic catalytic properties of bacteria for precise cancer treatment, showing a great promise. Oral administration is the most preferred and common route for a diverse range of drugs. However, most of the antitumor agents are not appropriate to be taken due to enzymatic degradation, acidic destruction, and poor penetration (Chen et al., 2018). Fan et al. (2018) reported an oral-administrated thermally sensitive therapeutic system applying noninvasive *E. coli* MG1655 as a vehicle for tumor therapy. Before biomineralized by gold nanoparticles (AuNPs), thermally sensitive programmable *E. coli* was modified with plasmids expressing tumor necrosis factor-α (TNF-α) to obtain TPB@Au, which could efficaciously protect AuNPs and TNF-α plasmids in the gut and transport to systemic circulation by microfold cell after oral administration. Subsequently, the expression of TNF-α at the tumor site could be triggered by the heat generated from AuNPs under NIR irradiation, leading to the inhibition of tumor cells. It has been reported that disturbed redox homeostasis will influence the growth of tumors (Sun et al., 2019). The levels of reactive nitrogen species (RNS) play a dual role, presenting pro-tumorigenic or antitumor at different intracellular concentrations, while tumor cells are directly killed under higher RNS concentrations. Zheng et al. (2018) constructed “charging” *E. coli* with photocatalyst to increase their metabolic activities. In this study, carbon nitride (C3N4) was linked to the surface of *E. coli* via electrostatic interactions carrying nitric oxide generation enzymes for photo-induced bacterial metabolite treatments. Under NIR irradiation, photoelectrons generated from C3N4 were transferred to bacteria to boost the enzymatic reduction of endogenous NO^3-^ to antitumor NO. The in vivo experiments showed that C3N4 loaded *E. coli* had a good inhibitory activity, with more than 70% inhibition.

### *Clostridium*-mediated cancer therapy

**FIGURE 6:**
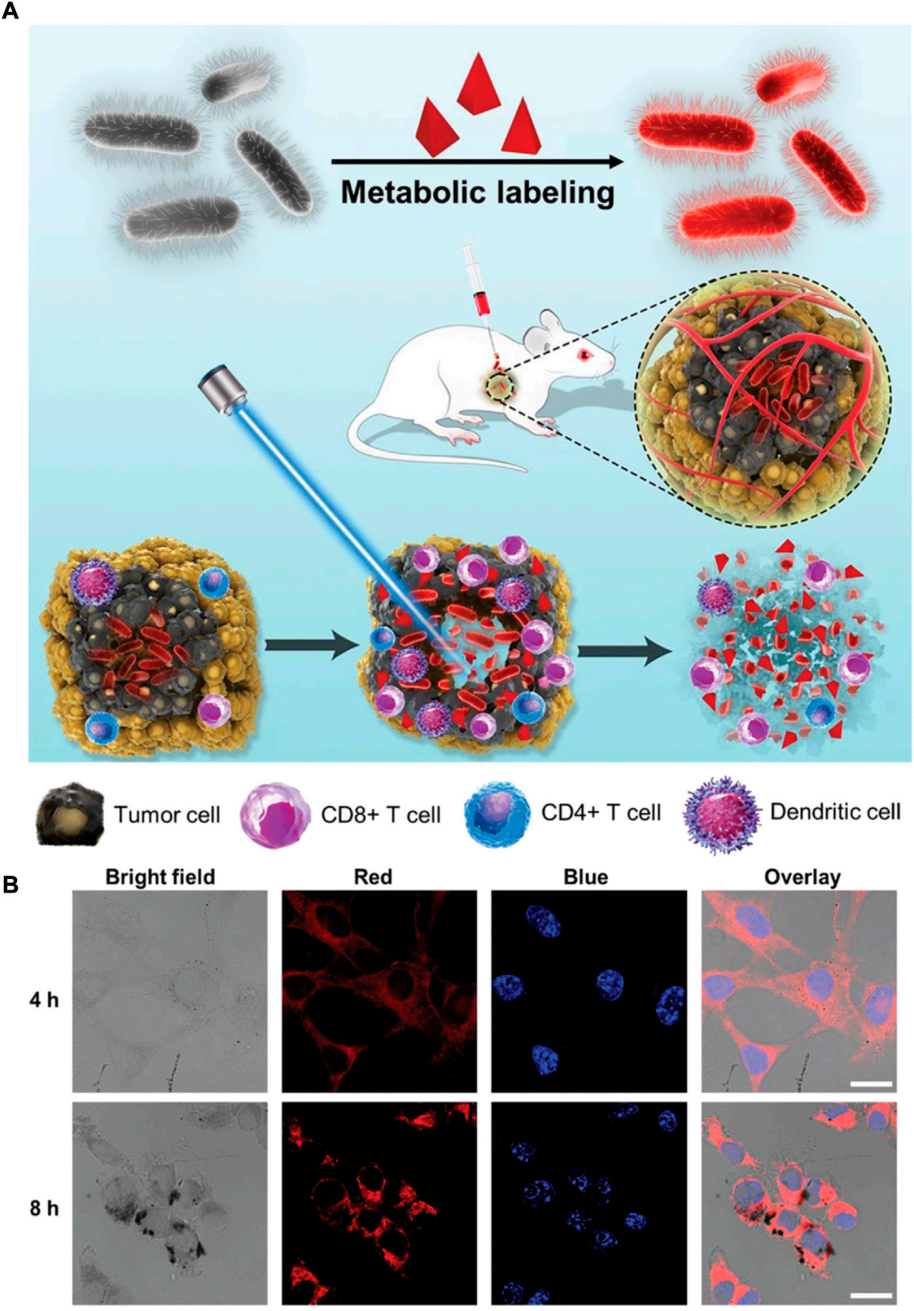
The construction of d-Ala-TPApy-coated *C. butyricum* for melanoma ablation. **(A)** Schematic depiction of d-Ala-TPApy-labeled bacteria and eradicating mechanism by labeled bacteria under NIR irradiation. **(B)** Representative confocal microscopy images of treating B16F10 cells with d-Ala-TPApy-labeled bacteria for 4 and 8 h. The blue and red fluorescence indicate cell nuclei and TPApy, respectively. Scale bar: 10 μm. Reproduced with permission from Shi et al. (2022). Copyright 2022, John Wiley and Sons.

*Clostridium* has been described as one of the largest prokaryotic genera and includes approximately 180 species (Dürre, 2014). Several species have enormous potential to act as desirable therapeutic agent delivery vehicles thanks to their natural ability to target and colonize in low-oxygen TME. *Clostridium* are anaerobic bacteria, which enables them to survive in a tumor site in contrast to normal tissues. To date, numerous subtypes of *Clostridium* have been used for antitumor therapy, such as *C. histolyticum*, *C. acetobutylicum*, *C. butyricum,* and *C. beijerinckii* (Van Mellaert et al., 2006). It is difficult to completely eradicate a malignant melanoma with conventional treatment, owing to their inherent properties of overall aggressiveness, complexity, and heterogeneity. Shi et al. (2022) proposed a new strategy, in which metabolic engineering labeled *C. butyricum* is able to ablate melanoma (Figure 6). In this study, d-alanine (d-Ala), a bacteria metabolic substrate of *C. butyricum*, was linked to AIE photosensitizer (TPApy) through click chemistry reaction to obtain d-Ala-TPApy. Engineered oncolytic *C. butyricum* was then prepared by d-Ala-TPApy metabolically attaching into bacterial peptidoglycan. After intratumoral injection, the bacteria could accumulate and proliferate in the tumor site, stimulate the immune TME, and ablate the hypoxia area. With the ablation of the tumor hypoxia region, the PS on the *C. butyricum* could exert photodynamic effects under light irradiation, thus further removing the tumor residue. A similar report was conducted by Park et al. (2017) in which branched gold nanoparticles (BGNPs) coated *Clostridium novyi*-NT (*C. novyi*-NT) spores were used for efficient computed tomography (CT) guided bacteriolytic tumor therapy. Electrostatic deposition methods were used to prepare BGNP-coated *C. novyi*-NT spores due to their facile procedure and relatively minimal toxicity. The in vivo results demonstrate that BGNP-coated *C. novyi*-NT spores display high therapeutic efficacy, which is consistent with native C. novyi-NT spores after intratumoral injection. This suggests that they have potential to act as delivery systems for combinational therapy. *Clostridium* is a promising hypoxia-targeted delivery platform that deserves further investigated, yet numerous issues and challenges still remain to be addressed.

### *Listeria*-mediated cancer therapy

**FIGURE 7:**
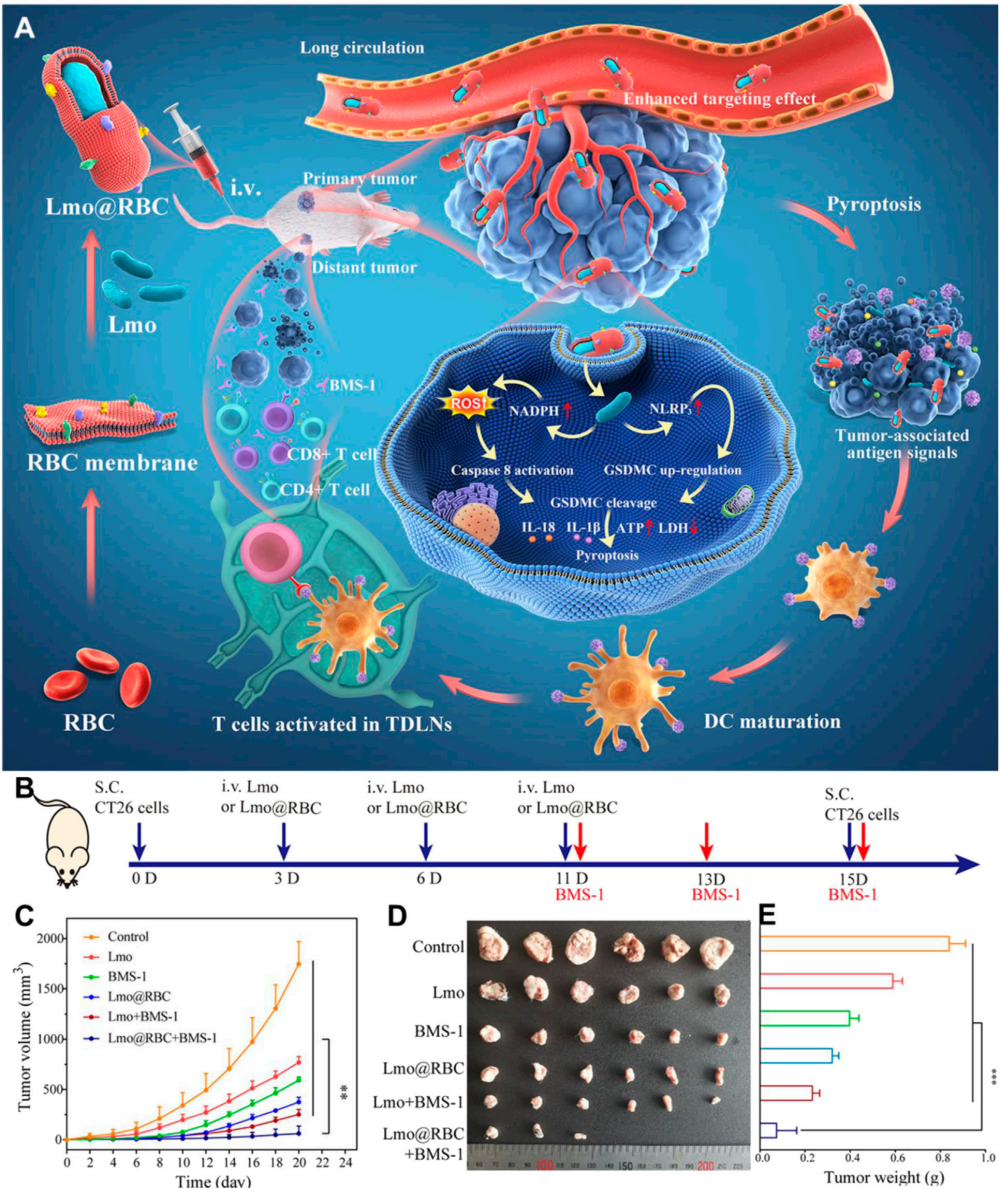
The use of Lmo@RBC to improve cancer immunotherapy. **(A)** Schematic illustration of the preparation and application of Lmo@RBC. **(B)** Schematic depiction of Lmo@RBC used for therapy in CT26 distant tumor-bearing mice. Primary tumor volume curve **(C)**, images of solid tumor **(D)**, and body weight **(E)** of mice intravenously administered with PBS, Lmo, BMS-1, Lmo@RBC, Lmo + BMS-1, or Lmo@RBC + BMS-1. Reproduced with permission from Liu et al. (2022). Copyright 2022, American Chemical Society.

*Listeria monocytogenes* is a facultative anaerobic bacterium that is one of the most commonly used vectors available for cancer immunotherapy. *Listeria* can infect people and produce various symptoms, such as meningitis, sepsis, and gastroenteritis. However, the characteristics that make *Listeria* pathogenic are also being engineered for delivery platforms in cancer therapy (Radoshevich and Cossart, 2018). In general, *Listeria* hijacks the host cell’s cytoskeleton dynamics to remain intracellular mobility and for cell-to-cell spread (Wood and Paterson, 2014). Compared to other microbial species, *Listeria* may treat deeper in tumors thanks to their inherent ability to evade phagolysosomal fusion and assist to deliver plasmid DNA into the cytoplasm (Hense et al., 2001). A variety of strategies have been used to engineer *Listeria* for cancer therapy, including the early report of a fluorescent or a bioluminescent gene loaded nanoparticles attaching on the *Listeria* surface to effectively express in solid human tumors (Akin et al., 2007). In vivo experiments have demonstrated the tumor-targeting properties of *Listeria*, and invasion and proliferation in solid tumors to ultimately deliver therapeutic genes. Thanks to these potential uses, *Listeria*-mediated cancer therapy has recently attracted much attention. Pancreatic cancer is the fourth leading cause of cancer deaths and has been called silent killer due to the metastasis initiating before the primary tumor diagnosed. Quispe-Tintaya et al. (2013) developed radioactive bacteria to selectively deliver the radioactivity to the metastases. They conjugated a radioactive anticancer radioisotope ^188^Rhenium to the cell wall of attenuated live *Listeria* via *Listeria*-binding antibodies. After intravenous injection into highly metastatic pancreatic tumor-bearing mice, the radioactive *Listeria* could deliver radioactivity to the metastases without harming normal tissues because of the efficient clear of bacteria in normal tissues by the immune system in contrasted to the immunosuppressed TME of metastases. The nontoxic radioactive *Listeria* applied to reduce or even eliminate pancreatic cancer recurrence and metastases have shown particular attractiveness for clinical development. To simplify the approach of preparing radioactive *Listeria*, Chandra et al. (2017) recently developed a new method in which 32-Phosphorus (^32^P) was directly incorporated into the living bacteria by applying ^32^P as a nutrient in contrast to conjugating ^188^Rhenium to bacteria by anti-Listeria antibodies. *Listeria*-^32^P was prepared by starvation of *Listeria* in saline before being cultured in ^32^P medium. According to the results, they found that *Listeria*-^32^P efficiently accumulated in tumors and metastases, and killed tumor cells though ionizing radiation induced by ^32^P and ROS induced by *Listeria*. Although *Listeria* shows a promising cancer vaccine platform, their rapid clearance and inevitable side effects have led to them being severely restricted in clinical application. Liu et al. (2022) coated attenuated *Listeria* (Lmo) with natural red blood cell (RBC) membranes to construct a new immunotherapy system (Lmo@RBC) (Figure 7). The prepared Lmo@RBC was not only protected from immune clearance in the immunosuppressive TME but also produced a low systemic inflammatory response due to the protection of RBC membrane. Benefiting from these advantages, the accumulation of Lmo@RBC was significantly increased after intravenous administration, resulting in enhanced ROS release and caspase-8 activation, and inducing cancer cell pyroptosis. Overall, Lmo@RBC, as a high biocompatibility platform and smart therapeutic drug for tumor pyrolysis, provides an opportunity for live bacteria vaccine.

### Photosynthetic bacteria-mediated cancer therapy

**FIGURE 8:**
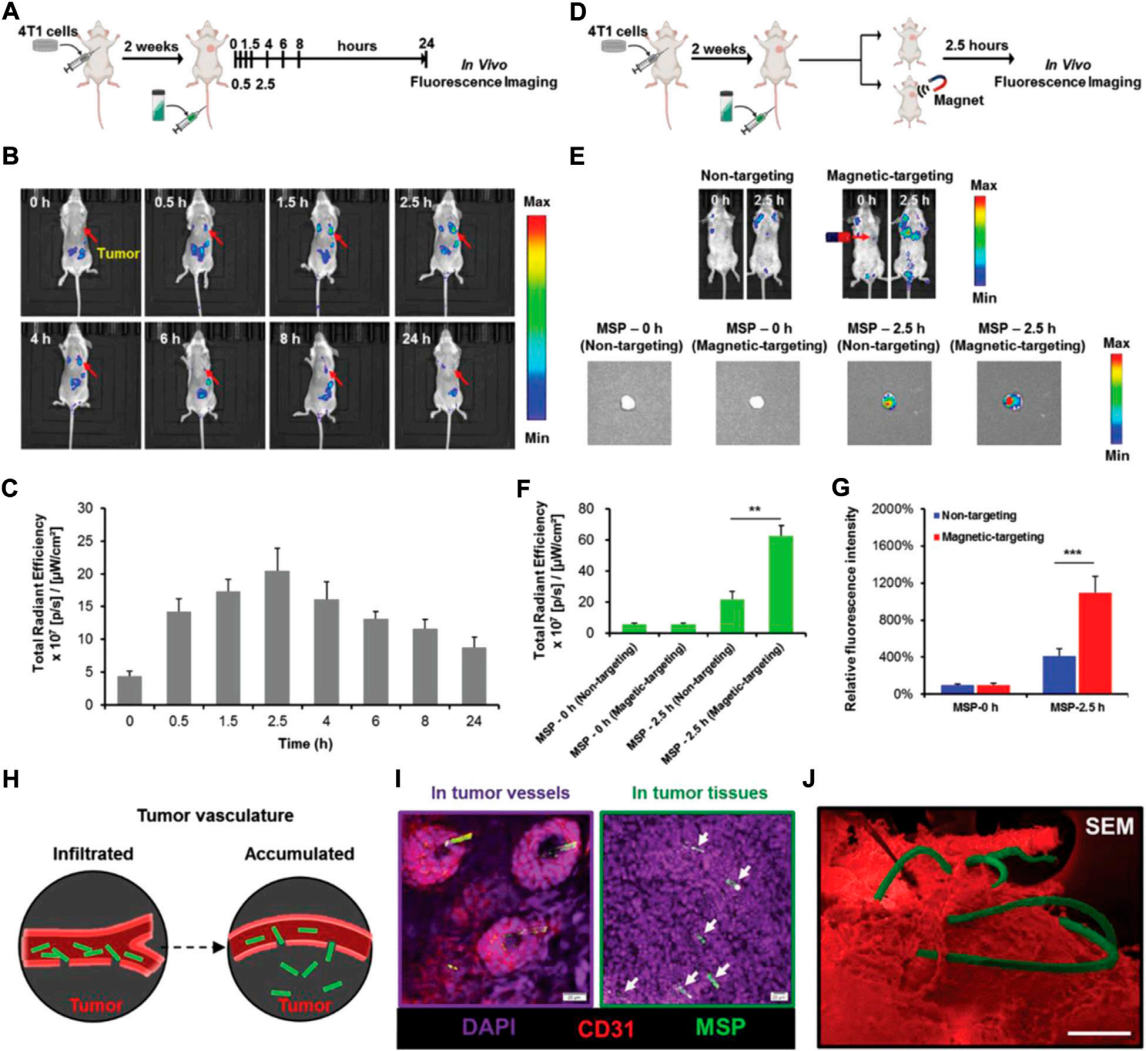
In vivo tumor accumulation of magnetic *S. platensis* under the external magnetic field. **(A)** Schematic depiction of the establishment of orthotopic 4T1-bearing mice model and the biodistribution of magnetic *S. platensis* in vivo. **(B)** In vivo time-dependent representative fluorescence imaging of 4T1-bearing mice post intravenous injection of magnetic *S. platensis* without external magnetic field. **(C)** Quantitative analysis of fluorescence intensities at 0.5, 1.5, 2.5, 4, 6, 8, and 24 h post i.v. injection of magnetic *S. platensis*. **(D)** Schematic depiction of orthotopic 4T1-bearing mice model and the targeting ability of magnetic *S. platensis* in vivo. **(E)** Representative fluorescence imaging of 4T1-bearing mice after intravenous injection of magnetic *S. platensis* at predetermined time. **(F)** Quantitative analysis of fluorescence intensities of a solid tumor. **(G)** Quantitative analysis of the mean fluorescent intensities of dissected tumors. **(H)** Schematic depiction of the infiltration and accumulation of magnetic *S. platensis* at tumor sites. **(I)** Tumor stained with DAPI (nuclei: purple) and CD31 (vessels: red). The green signals: magnetic *S. platensis*. Scale bar: 20 µm. **(J)** Representative SEM image of magnetic *S. platensis* (green) accumulating in tumor (red). Scale bar: 100 µm. Reproduced with permission from Zhong et al. (2020). Copyright 2020, John Wiley and Sons.

Photosynthetic bacteria (PSB) is a type of microbial species with photoenergy synthesis system that can generate organic substances under light exposure. A remarkable characteristic of PBS is that they can grow phototrophically instead of photosynthetically, and they can apply organic carbon compounds, hydrogen, and sulfides as hydrogenium donors to fix carbon dioxide for photosynthesis using light as an energy driver (Puyol et al., 2017). The light conversion properties of natural PSB are able to be exploited for cancer treatment. Yang et al. (2021) developed tumor-targeted optical immunotheranostics by applying NIR activatable non-pathogenic purple photosynthetic bacteria (PPSB) for PDT and PTT, together with photoacoustic imaging. Living PPSB have been used for cancer theranostics thanks to their multifunctionality and biocompatibility, using bio-optical-window first and second NIR light. The existing energy-transfer system of the light-harvesting nanocomplex in bacterial cell wall has made PPSB display a strong NIR-I to NIR-II fluorescent signal, excellent ROS generation, and contrasting photoacoustic signals, resulting in highly targeted tumor elimination and precisely tumor location. PSB can even be modified for the generation of oxygen, which may be used to improve the hypoxic TME. Liu et al. (2020) recently utilized active PSB (Synechococcus 7942, Syne) as an oxygen donor for tumor-targeted delivery of photosensitizers and in situ oxygen generation to increase the PDT efficacy. They assembled photosensitizer-coated nanoparticles (HSA/ICG) via intermolecular disulfide crosslinking. They then decorated them to the surface of Syne via amide bonds to obtain S/HSA/ICG, which possessed photosynthetic ability of bacteria and the theranostic capability of HSA/ICG. After intravenous injection, S/HSA/ICG exhibited effective accumulation in solid tumor and continuous generation of oxygen under light irradiation via photosynthesis. This led to remarkable relief of hypoxic TME and enhanced ROS production, and thereby finally eliminated the tumors. Similarly, Huo et al. (2020) designed and fabricated a kind of photosensitive and photosynthetic PSB to overcome the obstacle in tumor type-II PDT. The photosensitive bacteria were prepared by the hybridization of PSB and chlorine6 (ce6), which simultaneously sustained oxygen evolution by the bacteria and the generation of abundant ROS by the ce6 for malignant carcinoma destruction. The in vitro and in vivo results demonstrated that cascade oxygenation and photosensitization had successfully caused cytotoxicity and PDT. To reduce biological toxicity caused by longtime NIR irradiation and alleviate hypoxic TME, Chang et al. (2022) constructed a new exogenous irradiation-free bacteria-mediated PDT platform carrying persistent luminescence material (PLM). The blue-emitting PLM simultaneously activated PBS and verteporfin photosensitizer, which achieved continuous oxygen generation and singlet oxygen production without long-term external light irradiation. This resulted in the modulated hypoxic TME and enhanced PDT efficiency. To enhance the efficacy of hypoxia-targeted therapy, Zheng et al. (2021), successfully utilized *Rhodobacter johrii*, a species of living PSB, as hypoxia-targeted carriers with the capacities of NIR phototaxis for hypoxia cancer therapy. Interestingly, the living PSB exhibited photothermal properties through nonradiative relaxation pathways under NIR irradiation. Thus, the hypoxia targeting ability and photothermal property were integrated in PSB without complex modification in an all-in-one manner to achieve significant inhibition of tumor growth. Furthermore, natural PSB showed enhanced immune response to induce the T lymphocyte infiltration, which improved the efficiency in the tumor elimination. Although biohybrid microswimmers have been shown to perform actively targeted delivery to a tumor, the use of modified microswimmers in vivo treatments is still challenging. Zhong et al. (2020) proposed a photosynthetic biohybrid microswimmers system (PBNs) based on magnetic engineered *Spirulina platensis* (*S. platensis*) for tumor imaging and therapy. The engineered *S. platensis* was functionalized by superparamagnetic Fe_3_O_4_ nanoparticles using a dip-coating process, endowing them with precise locomotion and magnetic resonance imaging property (Figure 8). The results show that PBNs could act as an in situ oxygen generator in tumor tissue though photosynthesis, which improved hypoxic TME and thus enhanced the radiotherapy effectiveness. Moreover, the chlorophyll in the bacteria could be used as a photosensitizer, which generated ROS under light irradiation to achieve PDT. The combination of multimodal therapies could present an excellent tumor inhibition and provide a promising microrobotic platform for cancer theranostic applications.

### Other bacterial species-mediated cancer therapy

Hypoxic TME is the major driving force of bacterial motility for bacteria-mediated cancer therapy. Besides the aforementioned bacterial genera and species, other categories of bacteria have also been developed as candidates for delivering therapeutic agents in cancer. Magnetotactic bacteria, which are widely distributed in nature, are able to absorb iron ions from surrounding to synthesize magnetosomes with biomembrane coated. Recently, magnetic bacteria have been used with magnetism-driven tumor targeting for cancer diagnosis and treatment. Taherkhani et al. (2014) reported a new drug-delivery strategy that is composed of living *Magnetococcus marinus* MC-1 and drug-loaded liposomes through carbodiimide chemistry, showing effective delivery of active substances to solid tumors. Their in vitro results confirmed that the intrinsic bacterial motility and magnetic response of MC-1 were not changed by the attachment of a substantial number of liposomes. This engineered MC-1 combined magnetoerotaxis capability, native bacterial cytotoxicity, and therapeutic or diagnostic drugs transport, and was able to address the limitation of targeting and diffusion in the field of controlled release systems. Meanwhile, Felfoul et al. (2016) revealed that the magneto-aerotactic migration behavior of MC-1 can deliver drug-loaded liposomes to hypoxic areas of the tumor. After MC-1 carrying covalently bound drug-loaded liposomes were injected into severe combined immunodeficient beige mice, more than 55% of the bacteria were able to achieve to hypoxic areas of HCT116 colorectal xenografts under magnetic guiding.

*Lactic acid bacteria* (LAB) are common and abundant inhabitants of the human intestinal tract, and are very important for the long-term maintenance of health. Similar to the previous description of bacteria, the antitumor characteristics of LAB can be described as hypoxia-triggered motility, production of anticancer agents, stimulation of the host immune responses, and induction of cancer cell apoptosis. For instance, Shehata et al. (2019) confirmed that the extraction of different LAB strains shows anti-oxidant capabilities including free radical removal and H_2_O_2_ deposition. Selenium is an essential micronutrient for human health and has been shown to be associated with many different types of tumors. Yazdi et al. (2013) developed a new strategy of selenium nanoparticles (SeNPs) depositing on the surface of LAB though intracellular reduction of selenium ions for improving of immune responses. Their study showed that the level of some cytokines (e.g., IFN-γ and IL-17) significantly increased after oral administration of SeNPs attached bacteria, resulting in better prognosis in highly metastatic mouse mammary carcinoma. Reghu and Miyako (2022) recently reported a convenient approach of nanoparticle functionalized LAB for photothermal-induced cancer immunotherapy. *Bifidobacterium bifidum*, which are a commonly commercially produced LAB, were nanoengineered with ICG-encapsulating Cremophor EL nanoparticles via incubation and washing processes. The nanoengineered *Bifidobacterium* displayed unique optical properties, excellent biocompatibility, strong photothermal conversion, high tumor targeting, and powerful anticancer efficacy. Moreover, with the activation of immunological responses, the strong photothermal conversion of nanoengineered *Bifidobacterium* were able to be spatiotemporally evoked under NIR irradiation, effectively regressed the tumor in mice.

Apart from the previously mentioned applications, some other bacteria species have been explored for improving the therapeutic efficiency of cancer therapy. The excessive lactic acid in TME can promote therapeutic resistance of chemotherapy. Thus, the depletion of lactate in tumor tissue is a promising therapeutic approach to sensitize traditional tumor treatments. Wang et al. (2021) constructed an intelligent bioreactor (dfined as SO@MDH) by the combination of redox-sensitive DOX-loaded metal-organic framework MIL-101 nanoparticles (MDH) and *Shewanella oneidensis* MR-1 (SO) through an electrostatic interaction to sensitize chemotherapy. By taking advantage of the intrinsic TME tropism and respiration of bacteria, SO@MDH were able to actively target and accumulate in hypoxic regions of a tumor and metabolize the lactic acid in TME, simultaneously reducing Fe^3+^ ions in the MDH by accepting electrons from SO, leading to release of the DOX. With the reduction of Fe^3+^to Fe^2+^, the excess H_2_O_2_ in TME would promptly oxidized Fe^2+^ to Fe^3+^, which was beneficial to circularly catabolize lactic acid and significantly boost chemotherapy. Moreover, oral anticancer agent delivery has also attracted substantial attention because of its excellent compliance over other means of administration. Bacterial spores (the dormant life forms of bacteria) have been widely used as oral drug carriers to improve immunity levels. Yin et al. (2018) developed a novel *Bacillus*-based oral carrier for colon cancer treatment. Their in vitro and in vivo results showed that bacteria-mediated oral drug-delivery systems have a great potential for cancer therapy. Song et al. (2019) designed an oral autonomous nanoparticle generator that was based on *Bacillus cagulans* spores to overcome the multibiological barriers of gastrointestinal tract environment. These studies highlight the possibility of developing a bacterial spore-based oral drug-delivery system, showing a promising strategy for oral delivery of therapeutic agent.

## Conclusion and outlooks

Bacteria are considered to be promising candidates for cancer therapy thanks to their intrinsic properties, including hypoxia tropism, self-propelled motility, immunogenicity and their ability to be used as gene or drug carriers. Thus, various types of bacteria (e.g., *Salmonella*, *E. coli*, *Listeria*, and photosynthetic bacteria) have already been employed for solid and metastatic tumor treatment, with promising results. In addition, with the development of synthetic biology, complex gene circuits are able to integrate into bacterial cells for the expression of therapeutic proteins. Meanwhile, biomaterials have been widely applied to functionalize bacteria for targeted drug delivery, PTT, magnetothermal therapy and PDT, while promoting antitumor efficiency of synergistic cancer therapies. For example, 2-D materials or polymers attached on the surface of bacteria by simple mixing or stimuli-responsive covalent bonding result in not only increased drug loading capacities but can also benefit synergistic cancer therapy. However, bacteria-mediated cancer therapy has both advantages and disadvantages (Dang et al., 2001; Mengesha et al., 2007). This treatment has the advantage of high tumor-selectivity, easy genetic modification, and mobility to overcome the limitation of conventional therapies. Meanwhile, human trials have displayed that the interaction between bacteria and treatment drugs, the instability of genes, and biosafety require more consideration in bacteria-mediated cancer therapy. For example, systemic infection induced by bacteria carries a significant risk (Weerakkody and Witharana, 2019).

Although the successful regression of tumors has been displayed in experimental models, there are still some unsolved limitations related to potential host immune response, efficiency and accuracy of targeted delivery, and impeded bacteria self-reproduction. First, even if most of bacteria-mediated cancer therapy is based on attenuated strains, the components of bacteria cytoderm will cause innate immunity, provoking macrophage engulfment of bacteria, and sequentially decreasing the number of bacteria colonized in solid tumors. Second, like traditional therapeutics, most of the administrated bacteria are unavoidably eliminated by the reticuloendothelial system before arrival at the tumor site. In addition, the therapeutic drugs or biomaterials attached on the surface of bacteria do not increase with the proliferation of engineered bacteria, causing the dilution of effective concentration. Meanwhile, the bacterial self-propagation will lead to the therapeutic drugs or biomaterials detaching from the cell wall. Furthermore, while bacteria-mediated therapeutic agents have been applied in clinical trials, the potential biosafety of engineered bacteria at tumor regions should be properly addressed.

The development of synthetic biology and bionanotechnology has progressed rapidly in the last few decades, which combined with bacteria can provide further opportunities to address these limitations. First, to replace the current pathogenic bacteria, various avirulent probiotic or genetically engineered bacteria reducing inherent virulence without affecting TME targeting have been developed. For instance, *Lactococcus lactis*, as a naturally avirulent facultative anaerobic, is able to replace *Salmonella* to act as carriers targeting tumor hypoxic regions. Second, genetic modification also endows bacteria with response to various stimuli (including light, ultrasound, pH, chemical, and thermal stimuli), which further increases the in situ accumulation of bacteria at the tumor site. Third, nanoparticles, therapeutic agents, and liposomes functionalized on the cell walls of bacteria can improve their targeted accumulation and enhance their biocompatibility. At same time, surface functionalization by biomaterials mitigates the immunogenicity and toxicity of bacteria, which reduces the clearance by the immune system. Finally, engineered bacteria can synergize with conventional antitumor treatments for enhanced antitumor activities with reduction of adverse side effects in healthy tissues. In summary, bacteria-mediated cancer therapy is still in its infancy but is expected to be one of the essential treatments in the clinic, holding an immense potential to change the current forms of cancer therapy.
